# Radiological Perspectives in Congenital Sensorineural Hearing Loss: Insights from Cochlear Implant Candidates

**DOI:** 10.3390/jcm13247664

**Published:** 2024-12-16

**Authors:** Sabri Şirolu, Rauf Hamid, Seyfullah Halit Karagöz, Osman Aykan Kargın, Vefa Salt, Sevda Yener, Halide Çetin Kara, Emine Deniz Gözen, Serdar Arslan, Bora Korkmazer, Onur Tutar, Osman Kızılkılıç

**Affiliations:** 1 Sisli Hamidiye Etfal Training and Research Hospital, Radiology Clinic, University of Health Sciences, 34418 Istanbul, Türkiye; sabri.sirolu@gmail.com; 2 Department of Radiology, Cerrahpaşa Medical Faculty, İstanbul University-Cerrahpaşa, 34147 Istanbul, Türkiye; seyfullahhalitkaragoz@gmail.com (S.H.K.); vefasalt@gmail.com (V.S.); arslanserdar10@gmail.com (S.A.); borakorkmazer@gmail.com (B.K.); onurcpasa@gmail.com (O.T.); osmank@istanbul.edu.tr (O.K.); 3 Istanbul Physical Therapy and Rehabilitation Training and Research Hospital, Radiology Clinic, University of Health Sciences, 34147 Istanbul, Türkiye; aykankargin@gmail.com; 4 Department of Radiation Oncology, Cerrahpaşa Medical Faculty, İstanbul University-Cerrahpaşa, 34147 Istanbul, Türkiye; drsevda.kanat@gmail.com; 5 Department of Otorhinolaryngology, Cerrahpaşa Medical Faculty, İstanbul University-Cerrahpaşa, 34147 Istanbul, Türkiye; halidekara@gmail.com (H.Ç.K.); emine.gozen@iuc.edu.tr (E.D.G.)

**Keywords:** congenital sensorineural hearing loss, cochlear implantation, cochleovestibular malformations, computed tomography, magnetic resonance imaging, cochlear hypoplasia

## Abstract

**Objectives:** Congenital hearing loss is a significant health concern, with diverse etiologies encompassing cochlear and cochleovestibular pathologies. Preoperative radiological evaluation in cochlear implant candidates is pivotal for treatment planning. We aim to elucidate the spectrum of radiological findings in patients with congenital hearing loss undergoing cochlear implant assessment. **Methods:** An analysis included 389 sensorineural hearing loss (SNHL) patients who underwent cochlear implantation at a tertiary university hospital, of which 177 were congenital SNHL. Computed tomography (CT) and magnetic resonance imaging (MRI) data were meticulously assessed for diverse congenital pathologies, focusing on congenital malformations. **Results:** In the congenital SNHL group, comprising 177 patients (80 females and 97 males), congenital cochleovestibular malformations were evident in 56 ears of 29 cases. Different congenital cochleovestibular malformations, ranging from labyrinthine aplasia to isolated large vestibular aqueducts, were detected. Among the various anomalies, incomplete partitions and cochlear hypoplasia emerged as more frequent patterns. **Conclusions:** This study offers a comprehensive radiological analysis of congenital SNHL patients undergoing cochlear implantation, revealing a spectrum of anomalies. It demonstrates the diverse nature of anomalies affecting the external auditory canal, middle ear structures, and cochleovestibular system. These insights provide a deeper understanding of congenital SNHL and contribute to developing informed treatment strategies.

## 1. Introduction

Hearing loss is a reduced ability to perceive sounds that would otherwise be heard normally. It can be classified through various approaches. Congenital hearing loss refers to hearing loss present at birth or acquired shortly thereafter, while acquired hearing loss can occur at any stage of life in individuals with normal hearing after birth [1]. An alternative classification is based on the underlying pathogenesis. Conductive hearing loss (CHL) occurs when the intensity of sound waves is not fully transmitted to the cochlea. Sensorineural hearing loss (SNHL) results from damage to the inner ear structures, the cochlear nerve, or the central nervous system.

Congenital SNHL poses a significant public health concern, impacting approximately 1 in every 1000 births, with a higher incidence observed in specific patient groups, such as neonates requiring intensive care [2]. Early diagnosis and treatment are crucial because SNHL can significantly affect language and cognitive development, especially when cases remain undiagnosed for extended periods.

The primary cause of congenital SNHL is cochleovestibular malformations, which are rare developmental abnormalities that impact the inner ear structures responsible for hearing and balance [3]. These malformations encompass various types, each classified based on specific anatomical features that affect different components of the inner ear, such as the cochlea, vestibule, semicircular canals, and cochlear nerve, or a combination of these structures. Given their implications in patient management and surgical outcomes, correctly identifying and classifying these malformations is essential as correct classification allows for better surgical planning, such as the selection of cochlear or auditory brainstem implantation (ABI), and guides therapeutic decisions [4].

Several classification systems have been proposed, including the widely used system initially established by Jackler et al. [5] and further developed by Sennaroğlu and Saatçi [6] and Sennaroğlu and Bajin [4]. This classification system identifies eight primary types of cochleovestibular malformations: complete labyrinthine aplasia (LA), rudimentary otocyst, cochlear aplasia, lateral semicircular canal-vestibular dysplasia (LCVD), cochlear hypoplasia (CH), incomplete partitions (IP) (types 1-2-3), semicircular canal (SCC) malformations, and cochlear aperture anomalies. A large vestibular aqueduct (VA) is the abnormal dilation (≥1.5 mm, at the middle part) of the vestibular aqueduct, often accompanied by other malformations [7]. Each subtype presents unique anatomical features and hearing characteristics, necessitating customized approaches to cochlear implantation. For instance, specific major malformations, such as cochlear aplasia, cochlear nerve aplasia, and LA, are considered to be contraindications for surgery [8]. Moreover, several other malformations, including common cavity deformity and severe hypoplasia, elevate the risk of surgical complications [9].

Imaging methods are pivotal in accurately identifying and characterizing congenital malformations, which in turn facilitates more precise treatment planning and improves patient outcomes. Both high-resolution computed tomography (CT) and magnetic resonance imaging (MRI) provide essential information about inner ear anatomy that directly influences cochlear implant candidacy decisions. Appropriate imaging allows the surgeon to ascertain the presence of cochleae and cochlear nerves, detect malformations, evaluate the caliber of the cochlear nerves, and identify other anatomical factors that might impact surgical planning [10]. By combining the complementary advantages of CT and MRI, clinicians can perform comprehensive pre-operative evaluations that are critical for optimizing treatment outcomes in patients with congenital anomalies or acquired pathologies [11].

This study aims to evaluate the pre-operative CT and MRI scans of patients with congenital SNHL who have undergone cochlear implantation, with a primary focus on imaging-detectable malformations. The goal is to identify and categorize a spectrum of pathological radiological findings and contextualize them within the existing literature.

## 2. Materials and Methods

### 2.1. Study Group, Inclusion Criteria, and Exclusion Criteria

Our study evaluated 389 SNHL patients who underwent cochlear implantation at our university hospital over eight years. This study was approved by our institutional Ethics Committee (24637817-302.14.01-41492).

A total of 75 patients whose CT and MRI images were inaccessible and 30 patients whose available images did not follow the temporal bone protocol (such as brain CT/MRI) or were deemed unsuitable for evaluation (due to reasons such as motion artifacts) were excluded from the study.

**Figure 1:**
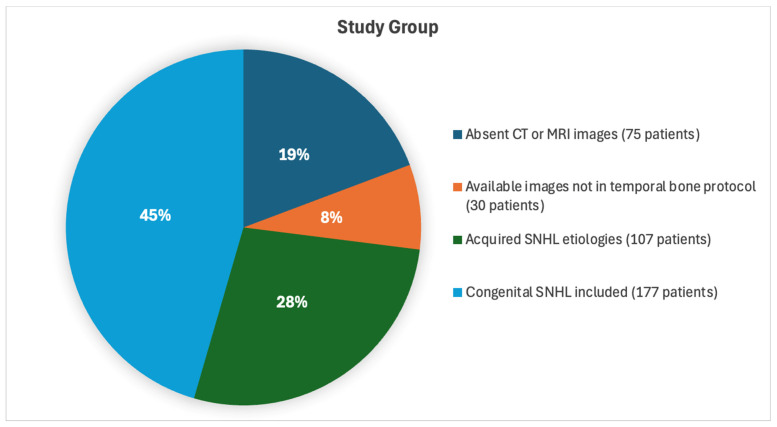
The pie chart represents the distribution of patients included in the study, outlining the process of inclusion and study design. It highlights the proportions of patients based on imaging availability and diagnostic categories. SNHL, sensorineural hearing loss; CT, computed tomography; MRI, magnetic resonance imaging.

Subjects diagnosed with hearing loss before one year of age who failed the neonatal hearing tests (177 subjects: 80 females, 97 males) were categorized as having congenital hearing loss and included in the study under this classification. The remaining 107 patients with acquired pathologies were grouped and evaluated separately as the control group (Figure 1).

### 2.2. Measurements and Statistical Analysis

All measurements and classifications were performed jointly by S.Ş. and V.S. In cases of disagreement, S.A. and O.K. were consulted for consensus.

Statistical analyses were conducted using the SPSS Statistics software (IBM Corp. Released 2021. IBM SPSS Statistics for Macintosh, Version 29.0. Armonk, NY, USA). Continuous variables were reported as means accompanied by standard deviations for normally distributed data. The median, minimum, and maximum values were reported for non-normally distributed data. Categorical variables were presented as frequencies or percentages. The baseline data were assessed using the Kolmogorov–Smirnov test. Distinctions between quantitative and categorical variables were examined using logistic regression in cases of non-normal data distribution. A significance threshold of *p* < 0.05 was used to determine statistical significance. *p*-values less than 0.05 were considered statistically significant.

### 2.3. Radiological Diagnostic Methods and Evaluation

#### 2.3.1. Computed Tomography (CT)

CT imaging was obtained on G. E. Revolution Evo 128 detector CT. The high-resolution CT images were obtained with 20 × 0.625 collimation and 0.8 mm thickness using 320 mAs and 120 kVp with ultra-thin image reconstruction using the high-resolution bone algorithm in the axial plane with 0.5 mm section thickness, 0.01 mm increments, and a FOV of 100, with a matrix size of 512 × 512. Theses isotropic image data were used to obtain coronal and sagittal reformatted images. The image interpretation was made using a 3D workstation.

Developmental anomalies in the external auditory canal were specifically evaluated, with stenosis defined as an external auditory canal (EAC) diameter of less than 4 mm [12]. In terms of middle ear structures, these were examined for congenital abnormalities that might affect the bony ossicles, the round window, and the oval window

**Figure 2:**
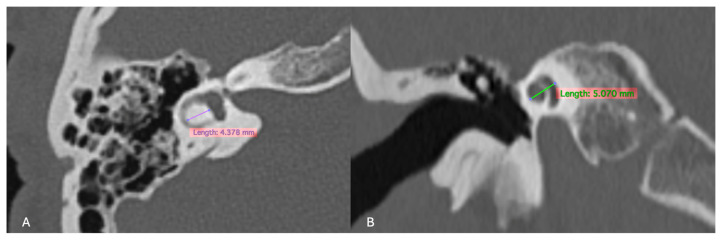
(**A**) Measurement of the LSCC (left semicircular canal) bony island in the axial section and (**B**) measurement of the height of the cochlea in the coronal reformatted section as described by Purcell et al. [13].

For the inner ear structures, we assessed the density of the otic capsule, the spiral structure, and the internal relationships, including the densities of the cochlea, vestibule, and semicircular canals. Cochlear malformations were classified according to the classification initially proposed by Jackler et al. [5] and later refined by Sennaroğlu and Saatçi [6]. Additionally, measurements of the cochlear height in the coronal section and the diameter of the bony island of the lateral semicircular canal (LSCC) in the axial section, as described by Purcell et al. [13], were taken for all patients to explore differences between congenital and acquired cases (Figure 2). Axial sections were aligned parallel to the LSCC, and coronal sections were defined as perpendicular to the axial sections. The VA was examined, and cases where the diameter exceeded 1.5 mm at its midpoint were identified as having a large VA.

Measurements of the internal acoustic canal (IAC) were conducted in the coronal plane, with an IAC narrower than 2 mm classified as stenosis [14]. Additionally, CT scans were employed to examine for any lesions at the cerebellopontine angle and within the brain parenchyma.

All patients had their internal carotid artery (ICA) and high jugular bulb assessed. Lateralized ICAs were identified when the lateral part crossed a line drawn through the midpoint of the basal turn of the cochlea [14]. It was defined as ICA dehiscence when the septum between the lateralized ICA and the tympanic cavity could not be distinguished. The criterion for the ‘high jugular bulb’ was the bulb’s dome extending beyond the IAC’s inferior border. It was defined as a jugular bulb dehiscence when the septum between the jugular bulb and the tympanic cavity could not be distinguished.

#### 2.3.2. Magnetic Resonance Imaging (MRI)

All MRI scans were obtained with Siemens Avanto 1.5 T (Erlangen, Germany) scanner with a 16-channel head coil.

The dimensions, morphologies, and signal intensities of labyrinthine structures were evaluated with special attention given to the presence of modiolus and interscalar septum, as these are significant in the differential diagnosis of incomplete partitions (IPs) and cochlear hypoplasia (CH). The facial, cochlear, and vestibular nerves, including their superior and inferior divisions, were also examined using axial, coronal, and sagittal oblique reformatted images. Heavily T2-weighted sequences, such as Constructive Interference in Steady State (CISS) and Fast Imaging Employing Steady-State Acquisition (FIESTA), were primarily utilized for this assessment. Cochlear nerve hypoplasia was diagnosed when the nerve’s thickness was found to be thinner than either the ipsilateral facial nerve or the contralateral cochlear nerve [4]. Based on this, cochlear nerves were classified as normal, hypoplastic, or aplastic/indiscernible.

In addition, the cerebellopontine angle and intracranial structures within the temporal MRI area were evaluated for any incidental or additional findings that could provide further insight into the pathogenesis of hearing loss.

## 3. Results

A total of 177 patients with congenital SNHL, diagnosed with hearing loss before the age of 1 or who failed the neonatal hearing tests, were evaluated. Of these patients, 80 were female and 97 were male, with a median age of 2 years (range 1–33 years). In the congenital SNHL group, 35 patients exhibited various abnormal imaging findings. Six of these patients had isolated large VA. The remaining 29 patients with additional findings are outlined in Table 1, while the remaining 142 patients showed no abnormality in radiological imaging.

CT scans were utilized to evaluate EAC pathologies. EAC stenosis (<4 mm) was observed in four ears of three patients, while borderline stenosis (4 mm) was detected in one ear. One particularly notable case involved a patient with bilateral EAC stenosis, with both sides measuring 3 mm. This patient also exhibited several additional findings, including a syndromic facial appearance, bilateral CH, bilateral semicircular canal hypoplasia, cleft palate, corpus callosum dysgenesis, interdigitation of gyri due to fenestration of falx cerebri, and hyperintensities on T2 and FLAIR sequences adjacent to the bilateral lateral ventricles. Another patient, a 1-year-old with congenital hearing loss, had EAC diameters of 3 mm on the right and 4 mm on the left, while a 4-year-old patient had an EAC diameter of 3.5 mm on the right. No additional anomalies were detected in these patients.

Four patients with a history of congenital SNHL exhibited malformations involving either the oval or the round window. In one case, bilateral aplasia was observed in all semicircular canals along with bilateral vestibular hypoplasia. The round window was visible in both ears, but the oval window was not evident on CT. In another case, where LA was detected in the left ear, the malleus and incus were evident, while the stapes, oval, and round windows were not detectable in the same ear. Two cases featuring bilateral IP-3 anomalies displayed bilateral malformed oval and round windows (Table 2).

Congenital cochleovestibular malformations, other than isolated large VA, were detected in 56 ears of 29 patients. These anomalies encompassed a spectrum of conditions, including LA, CH, IP-1, IP-2, IP-3, LCVD, and isolated lateral semicircular canal hypoplasia (LSCCH) in 1, 25, 5, 9, 4, 6, and 6 ears, respectively. No cochlear aplasia, rudimentary otocyst, or common cavity malformations were detected. While most patients exhibited bilateral findings, there were unique cases where two different anomalies were observed in both ears, and only one ear was affected. Table 1 comprehensively summarizes these malformations, including detailed patient-specific findings. Specific key observations and unique cases are highlighted in the text to underscore their clinical significance and rarity.

**Figure 3:**
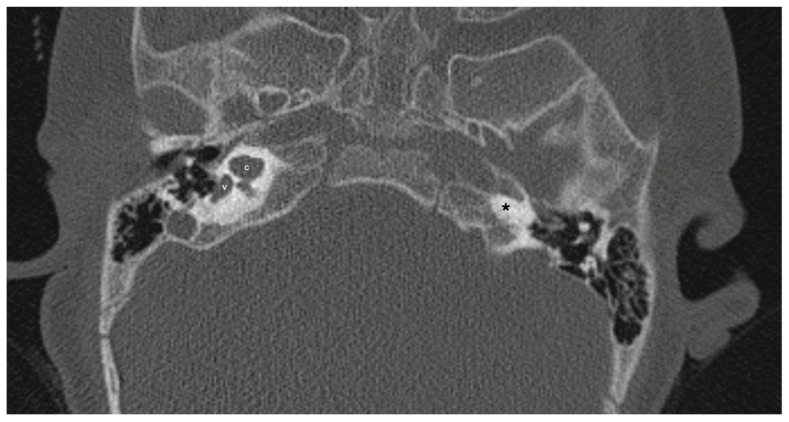
Patient with right-sided IP-1 (incomplete partition type-1) and left-sided LA (labyrinthine aplasia). On the right side, the cochlea (c) and the vestibule (v) are clearly differentiated. The cochlea has a near-normal size but lacks the entire modiolus and interscalar septa. None of the labyrinthine structures except the dense otic bone (*) can be seen on the left side.

**Figure 4:**
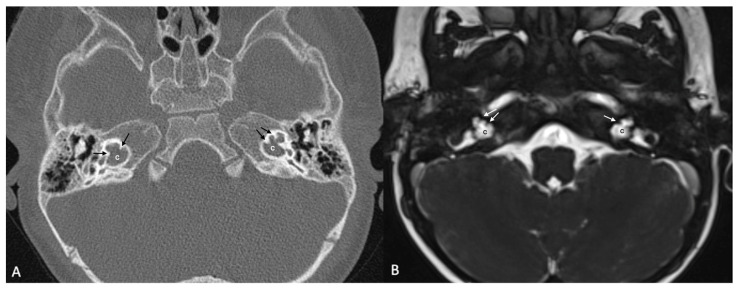
(**A**) CT and (**B**) MRI images of a case of bilateral IP-3 (incomplete partition type-3). Note that despite the relative preservation of the outer contour of both cochleae (c) and the presence of interscalar septae (black and white arrows), the inner structure is featureless and the modiolus is absent.

One patient exhibited IP-1 in the right ear and LA in the left ear. The right vestibule was hypoplastic, and the SCCs appeared as a single bud. The diameters of the right IAC and cochlear nerve were within normal limits. However, the stapes, oval and round windows, cochlea, vestibule, SCCs, IAC, and vestibulocochlear nerve could not be detected in the left ear. Cochlear implantation was performed in the right ear of this patient (Figure 3). Bilateral IP-3 anomalies were detected in two siblings. In these cases, the oval and round windows were also malformed. While both cochlear nerves of one sibling exhibited hypoplasia, the cochlear nerves of the other sibling were within normal limits as they were thicker than the facial nerves (Figure 4).

**Figure 5:**
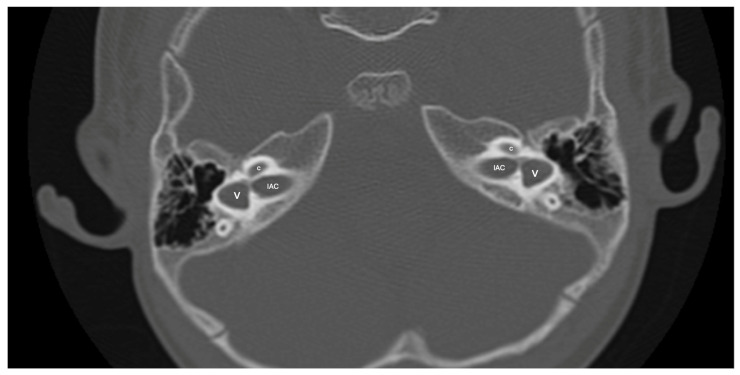
A case of bilateral lateral semicircular canal-vestibular dysplasia (LCVD). Both vestibules (v) are dilated and form a common cavity with the lateral semicircular canals (LSCC). The cochlea (c) can be normal (partially shown). IAC, internal acoustic canal.

LCVD was detected in six ears of three patients, while isolated LSCCH was detected in six ears of three patients. No additional malformations were detected in these patients’ cochleae or other inner ear structures (Figure 5). Cochlear nerve anomalies were observed in 11 patients with a history of congenital SNHL. Among these, seven had bilateral hypoplasia, two had isolated right cochlear nerve hypoplasia, one had isolated left cochlear nerve aplasia, and one had left cochlear nerve hypoplasia while the right cochlear nerve could not be distinguished. Within this group, only one patient showed no cochlear anomaly.

Eight patients exhibited large VA, while two patients had it unilaterally. VA could not be evaluated in 1 ear of a patient with labyrinthine aplasia. Additionally, one case demonstrated bilateral large VA and IP-2 (Mondini deformity). The remaining 6 patients did not have an additional cochleovestibular malformation detectable with imaging. Three patients were diagnosed with distal renal tubular acidosis (RTA), all of whom exhibited bilateral large VA as the sole imaging anomaly.

Measurements of cochlear height and the width of the LSCC bone island, as defined by Purcell et al. [13] in all patients. The patients were categorized into three groups: those with cochleovestibular anomalies (56 ears), patients with congenital SNHL but without detectable anomalies on imaging (298 ears), and those with acquired SNHL (214 ears). In the anomaly group, cochlear morphology in 4 ears was too distorted to measure the height, leaving 52 ears for evaluation. These ears showed significantly reduced cochlear height compared to the 512 ears without anomalies (*p* < 0.001). Additionally, the 298 ears with congenital hearing loss but without detectable anomalies had a lower mean cochlear height than the 214 ears with acquired hearing loss (*p* < 0.001). Of the 56 ears with congenital anomalies, 27 lacked detectable LSCC, leaving 28 ears available for evaluation. In the acquired SNHL group, LSCC assessability was compromised in 3 ears due to otosclerosis and trauma, allowing for the assessment of 211 ears. Notably, the LSCC bone island width was significantly lower in the anomaly group (28 ears) compared to the combined non-anomaly groups (509 ears) (*p* = 0.012). However, there was no significant difference in LSCC island width between the congenital SNHL group without anomalies and the acquired SNHL group. These findings suggest that cochlear height might be a more reliable indicator than LSCC bone island diameter for identifying congenital SNHL.

The evaluation of vascular structures was exclusively conducted using CT scans. Among the 242 CT scans reviewed, a high jugular bulb variation was identified in 62 cases (12.8%) out of the 484 ears examined. However, no instances of jugular bulb dehiscence were observed in any of these cases. A lateralized ICA was detected in 86 (17.8%) of the 484 ears evaluated. Notably, there was a single instance of ICA dehiscence in one ear, accompanied by a lateralized ICA in the contralateral ear for one patient.

Among the 177 patients diagnosed with congenital SNHL, nine patients (5.1%) exhibited pathological intracranial findings, while 17 patients (9.6%) had accompanying disorders. A notable case is a patient with a bilateral cochlear posterior semicircular canal and cochlear nerve hypoplasia who also had corpus callosum dysgenesis, falx cerebri agenesis, and periventricular white matter hyperintensities. Further details regarding these findings are provided in Table 3.

## 4. Discussion

This study aimed to delineate radiological findings in patients with congenital hearing loss and categorize malformation types. The analysis of these findings provides insights that are directly applicable to clinical and surgical decision-making, particularly for cochlear implant candidates.

Several algorithms exist for classifying congenital inner ear malformations. The current prevailing model, proposed by Jackler et al. [5] and refined by Sennaroğlu and Saatçi [6], is widely used. However, other models, such as those introduced by Phelps [15,16], Papsin et al. [17], and Adibelli et al. [18], have also been proposed.

Our study did not encounter any cases of cochlear aplasia, rudimentary otocyst, or common cavity malformations. Cochlear aplasia and rudimentary otocyst are contraindications for cochlear implantation [15]. Given that common cavity malformation presents a distinctive surgical challenge, the absence of these cases in our study suggests a tendency toward selective surgical approaches.

We identified congenital cochlear anomalies in 44 ears in total: 1 case of LA (2.3%), 5 cases of IP-1 (11.4%), 25 cases of CH (56.8%), 9 cases of IP-2 (20.5%), and 4 cases of IP-3 (9%). When vestibule and SCC anomalies were included, the rates varied as follows: 1 case of LA (1.8%), 5 cases of IP-1 (8.9%), 25 cases of CH (44.6%), 9 cases of IP-2 (16.1%), 4 cases of IP-3 (7.1%), 6 cases of LCVD (10.7%), and 6 cases of LSCCH (10.7%).

In a review by Joshi et al. [14], the reported frequencies of malformations among patients with congenital hearing loss were as follows: LA 1%, cochlear aplasia 3%, common cavity malformation 25%, IP-1 6%, CH 15%, and IP-2 50%. In this ranking, which did not include IP-3, half of the patients exhibited IP-2 malformation, and 15% had CH. The most significant difference between patients in our study was the prevalence of CH, which constituted the most common cochlear malformation at 56.6%, followed by IP-2 at 20.5%. In a review published by Sennaroğlu and Bajin [4], IPs accounted for 41% of all cochlear malformations (20% IP-1, 19% IP-2, 2% IP-3). Our results aligned more with the data presented by Sennaroğlu and Bajin regarding the frequency of IP-2 malformations. The disparities in these data can be attributed to different classification methods, variations in radiologist experience, and diverse patient populations across the studied centers.

Large VA is the most common inner ear malformation, frequently associated with other inner ear malformations [4]. However, the prevalence of large VA significantly varies across different studies. While early studies report rates as high as 84% [16], more recent studies have reported lower rates. In the series of Koesling et al., accompanying anomalies were found in 15 out of 28 ears with large VA [17]. In our patients, large VA was detected in 18 ears of subjects with a history of congenital hearing loss, and cochlear malformations were found in only 3 of those ears.

Another noteworthy observation is that hearing loss accompanying distal RTA often manifests with large VA as the sole imaging finding [19]. As the number of such patients increases within the overall patient population, the rates of malformations accompanying large VA also change. In our study, three individuals with a history of distal RTA were found to have a large VA.

In 2006, Purcell et al. [13] suggested that measurements of cochlear height and LSCC bone island width could serve as indicators of congenital malformations even in ears that appear morphologically normal. According to the data we obtained from this study, the following can be said:

- Cochlear height was significantly lower in patients with anomalies compared to those without, and it was also lower in patients with congenital hearing loss without anomalies compared to those with acquired hearing loss;
- LSCC bone island width was notably reduced in patients with anomalies compared to those without. However, for congenital SNHL patients without anomalies, the LSCC island width did not show a significant difference when compared to acquired SNHL patients;
- These findings suggest that cochlear height might be a more reliable predictor than LSCC bone island diameter for identifying congenital SNHL.

Since our study focused on cases involving cochlear implantation, patients ineligible for implantation due to contraindications were excluded from the study. Absolute contraindications for cochlear implantation included but were not limited to the absence of cochlea (whether normal or malformed) and/or cochlear nerve [15].

Two of our patients had an absolute contraindication in one ear, which was detectable through imaging, and implantation was carried out in the other ear. In a patient with bilateral CH, the right cochlear nerve was hypoplastic, whereas the left cochlear nerve could not be detected by imaging. In the other patient, an IP-1 anomaly was observed in the right ear, and LA was present in the left ear. While the right cochlear nerve of this patient displayed a normal caliber, the left cochlear nerve could not be detected. In both cases, implantation was carried out in the right ear due to contraindications against left-sided cochlear implantation.

Cochlear nerve hypoplasia/aplasia is often accompanied by cochleovestibular malformations, with some studies suggesting an association rate of up to 100% [20]. However, earlier studies reported a less frequent rate of cochlear malformation accompanying cochlear nerve hypoplasia [21]. This difference could be attributed to advancements in imaging technology, changes in the definitions of anomalies and cochlear nerve hypoplasia, or the differences in the patient populations included in the studies. Cochlear nerve hypoplasia was identified in 17 ears of our 11 patients, while cochlear nerve aplasia was detected in 2 ears. Only one patient with bilateral cochlear nerve hypoplasia showed no other detectable anomalies on imaging. In all other cases, patients exhibited cochlear malformations alongside cochlear nerve hypoplasia (CH in 10 ears, IP-1 in 2 ears, IP-2 in 2 ears, IP-3 in 2 ears, and LA in 1 ear).

Central nervous system (CNS) abnormalities in candidates for cochlear implants have been documented in the literature with varying frequencies and encompassing various pathologies. Digge et al. identified CNS findings in 4 out of 72 pediatric patients with bilateral SNHL. These findings included arachnoid cysts, scaphocephaly, leukodystrophy, and cystic encephalomalacia, with each condition observed in one patient [22]. In a group of 40 patients, Jallu et al. identified intrauterine cytomegalovirus (CMV) findings in one patient and an arachnoid cyst in another [23]. Our study identified CNS findings in 9 patients (5.1%) among 177 diagnosed with congenital SNHL, including arachnoid cysts, intracranial cystic lesions, white matter hyperintensities, cortical atrophy, polymicrogyria, and dysgenesis of the corpus callosum. Only one patient with inner ear malformation (bilateral cochlear, posterior semicircular canal, and cochlear nerve hypoplasia) demonstrated additional intracranial findings (corpus callosum dysgenesis, falx cerebri agenesis, and periventricular white matter hyperintensities). Other cases of intracranial pathologies were not associated with any inner ear malformations. Reporting these CNS findings is crucial because they play a significant role in predicting the outcomes of cochlear implants. As highlighted by Proctor et al. [24], CNS abnormalities may range from incidental findings to severe conditions that could drastically affect the prognosis. Severe abnormalities such as multicystic encephalomalacia may predict poor implant performance or general prognosis, potentially impacting clinical decisions regarding the candidacy and long-term effectiveness of the implants. Additionally, CNS imaging before implantation serves as a useful baseline, ensuring any changes or developments post-implantation can be tracked despite potential imaging artifacts caused by the implant itself.

## 5. Conclusions

In conclusion, this study demonstrates that pre-operative CT and MRI are indispensable tools for clinicians when assessing cochlear implant candidates with congenital SNHL. The detailed imaging findings enable precise classification of malformations, identification of contraindications, and anticipation of potential surgical challenges. Radiological evaluation not only helps in determining candidacy but also in optimizing post-operative outcomes by allowing for better surgical planning and more informed patient counseling. Future research should continue to focus on refining these imaging techniques and exploring their predictive value in larger and more diverse patient populations.

Our study had several limitations, primarily stemming from its retrospective nature. The variability in image quality due to differing protocols over the years and inconsistent patient file information posed challenges. Furthermore, focusing solely on cochlear implantation cases led to excluding mild cases treated without implants and severe cases where implants could not restore hearing, potentially skewing our statistics. Additionally, the control group in this study consisted of patients with acquired SNHL, which was included to maintain the study’s simplicity and focus. While this approach provided comparative insights, a control group comprising children with normal hearing would have allowed for the establishment of normative imaging values and a deeper understanding of cochlear and vestibular anatomy. Future research should aim to address this limitation through multicenter studies with larger datasets, including patients with normal hearing, and inclusive research encompassing all hearing loss presentations. Such efforts could lead to refined classifications and improved prognostic insights for congenital SNHL.

## Figures and Tables

**Table 1:** The list of patients with radiologically detected congenital malformations, including isolated large vestibular aqueduct. Side, implantation side; EAC, external acoustic canal, SCC, semicircular canal; LSCC, lateral semicircular canal; PSCC, posterior semicircular canal; SSCC, superior semicircular canal; IAC, internal acoustic canal; N, normal; BL, bilateral, R, right; L, left; LCVD, lateral semicircular canal-vestibular dysplasia; CH, cochlear hypoplasia; IP, incomplete partition.

				INNER EAR						CN and IAC	
Case No.	Age	Gender	Side	Cochlear Structure	Vestibule Morphology	SCC Morphology	Cochlear Height	LSCC Width	Vestibular Aqueduct (N: <1.5 mm)	Cochlear Nerve Diameter	IAC Diameter (N: >2 mm)
1	2	M	R	N	BL LCVD	BL LCVD	R: 4.8 L: 4.8	-	N	N	N
2	12	F	R	N	N	BL LSCC hypoplastic	R: 4.6 L: 5.0	R: 2.5 L: 2.1	N	N	N
3	1	M	R	N	BL LCVD	BL LCVD	R: 4.7 L: 4.5	-	N	N	N
4	2	F	L	BL CH	BL hypoplastic	BL aplastic	R: 3.3 L: 3.4	-	N	BL hypoplastic	N
5	1	M	R	N	N	N	R: 5.0 L: 5.1	R: 3.1 L: 3.5	N	BL hypoplastic	N
6	4	F	BL	BL CH	BL bud-shaped	BL PSCC hypoplastic, others absent	-	-	N	-	BL stenosis
7	2	M	R	R: CH	N	R LSCC hypoplastic	R: 5.0 L: 4.9	R: 1.9 L: 3.0	N	N	N
				L: N							
8	2	M	R	BL IP-1	BL enlarged	BL LSCC hypoplastic	-	R: 2.5 L: 1.3	N	BL hypoplastic	N
9	2	M	L	BL CH	BL LSCC and PSCC formed a common cavity with a vestibule	BL LSCC and PSCC formed a common cavity with a vestibule	R: 3.5 L: 3.6	-	N	N	N
10	3	F	R	BL CH	BL hypoplastic	BL aplastic	R: 3.9 R: 4.0	-	N	BL hypoplastic	N
11	2	F	L	BL CH	BL hypoplastic	BL aplastic	R: 4.2 L: 3.7	-	N	BL hypoplastic	BL stenosis
12	2	F	BL	N	BL LSCC and PSCC formed a common cavity with a vestibule	BL LSCC and PSCC formed a common cavity with a vestibule	R: 3.0 L: 3.7	-	N	N	N
13	5	M	BL	R: IP-2	N	N	R: 4.4 L: 4.5	R: 3.3 L: 3.0	N	N	N
				L: N							
14	1	F	R	BL IP-2	N	N	R: 4.2 L: 4.0	R: 2.8 L: 3.2	N	N	N
15	4	M	R	BL IP2	N	N	R: 4.9 L: 4.8	R: 3.8 L: 3.6	BL enlarged	R: hypoplastic	N
16	32	F	R	BL CH	N	N	R: 4.4 L: 4.4	R: 3.4 L: 3.8	N	N	N
17	9	F	R	BL CH	R: LSCC fused with vestibule/L: N	R: LSCC fused with vestibule/L: LSCC hypoplastic	R: 3.1 L: 3.6	R: - L: 1.2 mm	N	N	N
18	5	M	L	BL IP-2	BL LSCC and vestibule formed a common cavity	BL LSCC and vestibule formed a common cavity	R: 4.2 L: 4.8	-	N	R: hypoplastic	N
19	12	M	R	BL CH	R: enlarged/L: N	R: LSCC hypoplastic	R: 4.1 L: 4.1	R: 0.8 L: 3.0	N	N	N
						L: N					
20	3	F	L	BL CH	BL vestibule and SCCs formed a common cavity	BL vestibule and SCCs formed a common cavity	R: 3.1 L: 3.3	-	N	R: hypoplastic	N
										L: aplastic	
21	17	M	L	BL CH	N	BL PSCC hypoplastic	R: 4.2 L: 4.6	R: 5.1 L: 5.0	R: enlarged	BL hypoplastic	R: N
									L: N		L: stenosis
22	2	M	BL	BL IP3	N	N	R: 3.8 L: 4.0	R: 4.0 L: 4.5	N	BL hypoplastic	N
23	2	F	R	R: IP-1	R: hypoplastic/L: absent	R: hypoplastic (single bud)	-	-	R: N	R: N	R: N
				L: absent		L: absent			L: absent	L: aplastic	L: absent
24	2	M	R	BL IP-1	BL vestibule and LSCC formed a common cavity	BL vestibule and LSCC formed a common cavity/BL SSCC and PSCC enlarged	-	-	N	N	N
25	4	M	L	BL CH	N	N	R: 4.9 L: 4.5	R: 3.2 L: 2.9	N	N	N
26	4	M	R	BL IP-3	N	N	R: 4.1 L: 4.0	R: 4.5 L: 4.5	N	N	N
27	22	F	R	N	N	BL LSCC hypoplastic	R: 4.7 L: 4.9	R: 1.9 L: 2.1	N	N	N
28	2	M	BL	BL CH	BL vestibule and SCCs formed a common cavity	BL vestibule and SCCs formed a common cavity	R: 3.5 L: 3.7	-	N	N	N
29	2	F	R	BL IP-2	N	N	R: 3.5 L: 3.5	R: 3.5 L: 3.4	N	N	N

**Table 2:** Summary of oval and round window anomalies with accompanying malformations in congenital SNHL patients. SCC, semicircular canal; LA, labyrinthine aplasia; IP, incomplete partition.

Case No.	Age	Gender	Oval/Round Window Anomaly	Accompanying Anomalies
1	2	F	Bilateral oval window hypoplasia	Bilateral SCC and vestibular hypoplasia
2	2	F	Agenesis of oval and round windows and stapes in the left ear	Left sided LA and right sided IP-1.
3	2	E	Bilateral malformed oval and round windows	Bilateral IP-3, bilateral cochlear nerve hypoplasia
4	4	E	Bilateral malformed oval and round windows	Bilateral IP-3

**Table 3:** Summary of patients with abnormal intracranial findings on imaging and/or accompanying diseases. RTA, renal tubular acidosis; CMV, cytomegalovirus; NMR, neuromuscular retardation.

Case No.	Age	Gender	Intracranial Pathology	Accompanying Diseases
1	25	F	White matter hyperintensities	-
2	1	F	-	RTA
3	2	F	-	RTA
4	4	F	-	Microcephaly, ventricular septal defect, epilepsy, renal agenesis
5	2	M	-	Silver-Russel syndrome, microcephaly, epilepsy, inguinal hernia
6	3	F	-	Charge syndrome, left eye coloboma, cleft palate
7	2	F	-	Right eye primary hyperplastic vitreous, cleft palate
8	1	M	Arachnoid cyst in temporal lobe	-
9	1	F	-	Charge syndrome, esophageal atresia
10	2	M	Bilateral cortical atrophy of occipital lobes, white matter hyperintensities, enlargement of occipital horns of lateral ventricles (sequela of hypoglycemia), coarse calcifications in parietal white matter	Epilepsy, microcephaly, cerebral palsy
11	1	F	Thinning of the corpus callosum, white matter hyperintensities, bilateral enlargement of lateral ventricles, band heterotopia, bilateral lissencephaly, bilateral frontal and parietal polymicrogyria	Congenital CMV infection
12	3	F	-	Hirschprung disease, Waardenburg syndrome
13	17	M	Corpus callosum dysgenesis, falx cerebri agenesis, periventricular white matter hyperintensities	Cleft palate
14	2	M	-	Waardenburg syndrome
15	4	M	-	NMR
16	3	F	Arachnoid cyst in the left cerebellar hemisphere	NMR
17	2	M	Arachnoid cyst in the left temporal pole	-
18	2	M	Hydrocephalus, atrophy of corpus callosum, cerebellar vermis hypoplasia, hypomyelination	Tracheoesophageal fistula, patent ductus arteriosus
19	4	M	Arachnoid cyst in the right temporal pole	-

## Data Availability

The data presented in this study are available on request from the corresponding author.
